# Recombinant Human Thymosin β4 Attenuates Endotoxemia-Induced ALI and EAE by Suppressing Inflammatory and Oxidative Responses

**DOI:** 10.3390/biom16060766

**Published:** 2026-05-22

**Authors:** Yumeng Ye, Xuefeng Yang, Ying Liu, Jingshuo Zhao, Tongtong Chen, Yujie Xing, Hongyan Zuo, Yanhui Hao, Yang Li

**Affiliations:** 1 Beijing Institute of Radiation Medicine, Beijing 100850, China; yym18647444488@163.com (Y.Y.); 15534803600@163.com (X.Y.); lllyyyy8102@163.com (Y.L.); 13363723271@163.com (J.Z.); 15900252487@163.com (T.C.); 15075279290@163.com (Y.X.); zuohy2005@126.com (H.Z.); 2 Department of Pathology, Chengde Medical University, Chengde 067000, China

**Keywords:** endotoxemia, thymosin β4, cytokine storm, oxidative stress, NF-κB, endotoxemia-associated encephalopathy, lipopolysaccharide, macrophages

## Abstract

Endotoxemia represents a life-threatening clinical disorder driven by an aberrant host immune response to pathogenic infection, often resulting in severe multiple organ dysfunction. Among its most devastating complications are acute lung injury (ALI) and endotoxemia-associated encephalopathy (EAE), both of which are associated with elevated mortality and currently lack effective targeted interventions. This study evaluated the therapeutic efficacy and underlying molecular mechanisms of recombinant human thymosin β4 (rhTβ4) in a murine model of lipopolysaccharide (LPS)-induced endotoxemia. Our results showed that treatment with rhTβ4 markedly enhanced survival rates and diminished the systemic overproduction of diverse proinflammatory cytokines and chemokines in endotoxemic mice. These systemic protective actions were achieved through the inhibition of the TLR4/NF-κB signaling cascade, the reduction in M1 macrophage polarization, and the simultaneous alleviation of mitochondrial impairment and oxidative stress. Moreover, rhTβ4 treatment significantly rescued EAE-related cognitive deficits and attenuated neuronal damage, primarily through the suppression of neuroinflammation and microglial overactivation. Integrative transcriptomic profiling and functional assays identified lysophosphatidic acid receptor 3 (LPAR3) as an important contributor, suggesting that rhTβ4 suppresses microglial-mediated neurotoxicity at least in part through LPAR3 downregulation. In conclusion, rhTβ4 confers robust multi-organ protection against endotoxemic injury by orchestrating the inhibition of systemic and central neuroinflammatory cascades, positioning it as a promising candidate for the treatment of endotoxemia-induced ALI and EAE.

## 1. Introduction

Endotoxemia is a life-threatening disorder characterized by organ dysfunction arising from an aberrant host response to endotoxin exposure and continues to represent a major clinical challenge in critical care, associated with elevated mortality and persistent long-term morbidity across the globe [1]. Although substantial advancements in supportive care strategies and antimicrobial therapeutic regimens, the development of effective, targeted interventions for endotoxemia has proven largely unsuccessful to date. Its underlying pathogenesis, which involves uncontrolled inflammatory cascades [2], immune suppression or dysregulation [3], and severe metabolic disturbances [4], is very complex and multifactorial; hence, there is an urgent need to develop new therapies.

The uncontrolled and systemic hyperinflammatory response, which is clinically known as a cytokine storm, serves as the critical pathological trigger in endotoxemia, characterized by the excessive production of the main pro-inflammatory mediators, including TNF-α, IL-6, and IL-1β [5,6]. This deleterious cascade disrupts endothelial integrity and microcirculatory homeostasis, ultimately culminating in multiple organ failure. Pulmonary and cerebral tissues are the most vulnerable among the affected systems. The lungs are the most frequently affected organ, and acute lung injury (ALI) and its more severe variant, acute respiratory distress syndrome (ARDS), are the leading causes of death [7,8,9]. These conditions are characterized by alveolar–capillary barrier disruption, pulmonary edema, and robust immune cell infiltration, with lipopolysaccharide (LPS) serving as a key pathogenic driver. Macrophages play a central role in this process. Upon activation, they produce abundant proinflammatory cytokines, chemokines, and cytotoxic mediators that amplify tissue damage. Concurrently, endotoxemia-associated encephalopathy (EAE) drives persistent cognitive deficits and poor long-term prognosis, primarily driven by neuroinflammation and microglial overactivation [10]. As the brain’s resident macrophages, microglia are the first responders to inflammatory stimuli [11]. Their activation via pattern recognition receptors initiates a cascade of pro-inflammatory signals, which in turn recruits peripheral immune cells, including T lymphocytes, through chemokine release and vascular adhesion modulation [12,13]. Given the parallel and organ-specific mechanisms underlying lung and brain injury, a dual-action therapeutic strategy represents an essential yet underexplored approach for effective intervention in endotoxemia.

Toll-like receptor 4 (TLR4) functions as a key sensor responsible for recognizing LPS, a major component of the outer membrane of Gram-negative bacteria, and its activation represents a critical early event in the induction of cytokine storm during systemic inflammation [14]. Upon binding to LPS, TLR4 initiates intracellular signaling cascades that ultimately converge on the activation of nuclear factor-kappa B (NF-κB), which further promotes the transcription and release of a wide range of pro-inflammatory cytokines and reactive oxygen species (ROS) [15]. Due to its ability to faithfully recapitulate the systemic inflammatory response and subsequent organ injury observed in clinical sepsis, the LPS-induced mouse model of endotoxemia has been widely adopted as a reliable experimental platform for investigating inflammatory mechanisms and evaluating potential interventions [16]. Because of its pivotal part in controlling inflammatory genes, the TLR4/NF-kB axis is a tempting target in anti-inflammatory treatment. However, traditional methods based on neutralization of individual pro-inflammatory cytokines have had limited effectiveness in clinical practice, which makes it important to consider targeting upstream regulatory pathways instead of individual downstream mediators [17]. Even though the identification of small-molecule TLR4 antagonists has remained difficult, peptides have been identified as a potential therapeutic approach because of their high specificity, good safety, and ability to intervene in the main protein–protein interactions in the TLR4 signaling complex [18]. Although the importance of TLR4 as a primary trigger of the inflammatory cascade in endotoxemia is well-documented, strategies aimed at targeting this pathway have not been clinically translated into success, partly owing to the lack of consideration of multi-organ protection and the complexity of cellular response. In particular, the ability of peptide-based therapies to simultaneously reduce pulmonary and cerebral damage, along with systematic changes in macrophage and microglia numbers, has not been sufficiently explored. We hypothesized that our recently developed peptide, a clinically safe [19] modified variant of human Thymosin beta-4 (rhTβ4), would be able to suppress endotoxemia-related injury by suppressing both cytokine storm and oxidative stress by inhibiting the TLR4/NF-κB pathway. To validate this hypothesis, we employed both in vitro systems, namely, LPS-stimulated RAW264.7 macrophages and BV2 microglial cells, and the in vivo system of murine LPS-induced endotoxemia to evaluate the therapeutic efficacy and underlying molecular mechanisms of rhTβ4. Our findings demonstrate that rhTβ4 exerts potent anti-inflammatory and antioxidant effects through modulation of the TLR4/NF-κB pathway and downregulation of lysophosphatidic acid receptor 3 (LPAR3), thereby attenuating inflammation and conferring dual protection against lung and brain injury. These findings place rhTβ4 as a potential multi-organ protector and justify its continued development as a treatment option for endotoxemia.

## 2. Materials and Methods

### 2.1. Experimental Animals

In this study, a total of 75 male C57BL/6N mice (20–22 g) and 45 female C57BL/6N mice (18–20 g) were used, all obtained from Beijing Viton Lihua Laboratory Animal Technology Co., Ltd. (Beijing, China). At the start of the trial, all mice were 8 weeks old. Animals were maintained at the Experimental Animal Center of the Academy of Military Medical Sciences, under a specific pathogen-free environment with a temperature of 22 ± 1 °C, relative humidity of 60%, and a 12/12-h light-dark cycle (lights on from 7:00 a.m. to 7:00 p.m.). No more than five animals were housed per cage. Mice were humanely euthanized either upon reaching predefined humane endpoints or at the conclusion of the experiments. Humane endpoints were defined to include, but were not limited to, a body weight loss exceeding 15%, inability to eat or drink, and overt signs of distress or pain. In the present study, no mice reached the predefined humane endpoints before the end of the experiments. The death of the mice was verified by the lack of respiration, heartbeat, and corneal reflex. All experimental procedures were approved by the Institutional Animal Care and Use Committee of the Academy of Military Medical Sciences (Approval No. IACUC-DWZX-2024-P697).

### 2.2. Cell Culture

RAW264.7, RLE-6TN, and BV2 cells, purchased from Wuhan Pricella Biotechnology Co., Ltd. (Wuhan, China), were cultured in DMEM (Gibco, C11885500BT, Waltham, MA, USA) supplemented with 10% fetal bovine serum (Vazyme, F101-01, Nanjing, China). The cells were incubated at 37 °C in a humidified environment containing 5% CO_2_. The culture medium was routinely replaced every two days.

### 2.3. Drugs

rhTβ4 was produced by Beijing Northland Biotechnology Co., Ltd. (Beijing, China) and passed its main quality index tests, achieving 100% purity via electrophoresis, >98% purity via HPLC, biological activity of 1.67, and endotoxin content < 10 EU/branch, batch number: C-20240801.

### 2.4. LPS Treatment

For the pharmacodynamic experiments in vitro, RAW264.7 cells, RLE-6TN cells, and BV2 microglia were treated with LPS (Sigma, L2630, St. Louis, MO, USA) at a final concentration of 500 ng/mL. Moreover, two different LPS doses were selected based on the distinct pathophysiological features of each model, as supported by established literature. For the ALI model, a dose of 10 mg/kg was used to induce robust and reproducible pulmonary inflammation, which is well-established as a standard for evaluating anti-inflammatory interventions [20]. For the EAE model, a lower dose of 5 mg/kg was chosen to induce a sustained systemic inflammatory response with manageable mortality, thereby allowing a sufficient time window to observe neuroinflammatory changes [21,22].

### 2.5. rhTβ4 Administration

The original solution (0.1 mg/mL) was prepared by adding 0.1 mg of rhTβ4 lyophilized powder to 1 mL phosphate-buffered saline (PBS). The drug was prepared on the day of each experiment.

In the LPS + rhTβ4 group, rhTβ4 (5 µg/kg, i.p.) was administered 30 min after LPS injection and then once daily for 3 consecutive days. Control mice received an equivalent volume of PBS on the same schedule.

### 2.6. Survival Assays

The survival of the LPS-induced mice was recorded daily for 72 h. The survival experiment was performed with 10 mice in each group. The time of death was recorded, and the death rate and median survival time were calculated.

### 2.7. Cytokine Detection

Mouse blood was collected and centrifuged at 3500 rpm for 5 min at 4 °C. The separated serum was then utilized for cytokine detection in both serum and tissue samples. The right lung from five mice in each group was cryopreserved, weighed, and combined with 1:10 RIPA lysate, then homogenized. Supernatants were collected after centrifugation. Protein concentrations were evaluated and analyzed by radioimmunoassay.

RIA was selected for cytokine quantification because it offers superior sensitivity for detecting low-abundance cytokines, which is particularly important for certain cytokines present at extremely low concentrations, and because it allows for the detection of cytokines and chemokines from small sample volumes. Briefly, the sample was added to the sample tube. Then, 100 μL and 200 μL of buffer were added to the non-specific binding (NSB) and standard (S0) tubes, respectively. Then, 100 μL of the standard was added to S0, and 100 μL of antibody was added to the other tubes. The radioactive cytokine marker I^125^ (100 μL) was added to all tubes, mixed, and placed at 4 °C for 24 h. Subsequently, 500 μL of secondary antibody separation agent was added to each tube and centrifuged for 25 min. The precipitation radioactive energy (cpm) value was recorded, detecting TNF-α, IL-1β, IL-6, IL-4, IL-13, IL-18, IFNγ, Ccl2, Ccl3, Ccl7, CXCL10, S100β, and NSE (all radioimmunoassay kits were provided by Furey Runze Biotechnology Co., Ltd., Beijing, China).

### 2.8. Flow Cytometry

Cells treated with LPS and rhTβ4 for 24 h were subsequently stained in DMEM for 30 min (37 °C, 5% CO_2_) with one of the following probes: DCFH (Beyotime, S0033S, Shanghai, China), MitoSOX (MCE, HY-D1055, Monmouth Junction, NJ, USA), Annexin V-FITC (Beyotime, C1062L), or a combination of MitoTracker Red CMXRos (Beyotime, C1035, Shanghai, China) and MitoTracker Green (Beyotime, Shanghai, C1048). Following staining, cell pellets were collected, washed with PBS, and resuspended. Flow cytometry was carried out on a FACSCanto II (BD, Franklin Lakes, NJ, USA).

Single cells were prepared as described before [23] and resuspended in PBS. Cells were immunostained with phycoerythrin (PE)/Cyanine7-conjugated anti-mouse CD45 (BioLegend, 157206, San Diego, CA, USA), PE-conjugated anti-mouse/human CD11b (101280, BioLegend, San Diego, CA, USA), and FITC-conjugated anti-mouse F4/80 (123108, BioLegend, San Diego, CA, USA). FACS Diva Software (BD, v8.0.1) was used to analyze the cells on a BD FACSCanto II flow cytometer (BD, Franklin Lakes, NJ, USA). Macrophages were defined as CD45^+^CD11b^+^F4/80^+^.

### 2.9. Neurobehavioral Testing

Behavioral tests were performed at 7 and 14 d after the first LPS/rhTβ4 injection, as illustrated in the experimental timeline. Mice were randomly assigned to three groups: Control, LPS, and LPS + rhTβ4 (*n* = 10 per group). On each testing day, the Y-maze spontaneous alternation test was performed first, followed by the NORT, with at least 4 h between the two tests to minimize carry-over effects. All tests were conducted during the light phase in a quiet, dimly lit room. Mice were acclimatized to the testing room for at least 30 min before each test.

Novel object recognition test (NORT): NORT was employed to evaluate short-term declarative memory. The test comprised a 5 min acquisition phase where mice were allowed to explore two identical objects, followed by a 4 h inter-trial interval. During the 5 min test phase, one familiar object was substituted with a novel one, and exploration time for each object was recorded by an investigator blinded to the experimental groups. Exploration behavior was defined as sniffing or whisking directed toward the object. The discrimination index (DI) for the novel object was determined using the formula: DI = [tN/(tN + tF)] × 100, where tN and tF denote time spent exploring the novel and familiar objects, respectively.

Y maze: A Y-maze test was performed to evaluate spatial working memory using the spontaneous alternation paradigm. Three identical arms (30 cm × 8 cm × 15 cm), named A, B, and C, comprised the maze. All the mice were first introduced into arm A and allowed to freely explore the three arms for 8 min. Entry into an arm was recorded when all four paws were inside an arm. Alternation was considered to be successive visits to three distinct arms. The percentage of spontaneous alternation was determined as follows: [number of alternations/(total arm entries − 2)] × 100. In order to remove the olfactory cues, the maze was cleaned using 70% ethanol between the trials.

### 2.10. Tissue Isolation

After anesthesia was administered via intraperitoneal injections of pentobarbital sodium (five mice per group), venous blood was collected and centrifuged to obtain serum for peripheral hemogram examination and detection of cytokines. The left cerebrum and left lung were fixed in formalin (Sinopharm Chemical Reagent Co., Ltd., Shanghai, China) and used as a specimen for pathological analysis, while the right cerebrum and right lung were snap-frozen in liquid nitrogen and kept for future radioimmunoassays and Western blots. Fixed tissues were stained with HE.

### 2.11. Hematoxylin-Eosin (HE) Staining

After fixation with 4% paraformaldehyde for 24 h, lung and brain tissues were embedded in paraffin and sectioned coronally at 3 μm by a microtome (Leica, Wetzlar, Germany). The sections were stained with hematoxylin after deparaffinization with xylene and rehydration with a graded ethanol series (100%, 95%, 75% and distilled water) to stain nuclei, washed with running tap water (10 min), and counterstained with eosin (2 min) to label cytoplasmic elements. Then, the sections were dehydrated in ascending grades of ethanol (75 percent, 95 percent, and pure alcohol), cleared in xylene, and coated with neutral resin. The histological analysis was performed using a light microscope (Olympus, Tokyo, Japan), and images of at least five random fields per section were taken.

### 2.12. Nissl Staining

To evaluate neuronal morphology in brain tissues, Nissl staining was conducted. Coronal brain sections were incubated with 0.1% Toluidine Blue solution (BL999A, Biosharp, Hefei, China) for 10 min at room temperature. After a brief rinse in distilled water, the sections were dehydrated through graded ethanol (75%, 95%, and absolute), cleared in xylene (two changes: 5 min and 10 min, respectively), and mounted with neutral resin. The stained sections were then examined under a SlideViewer microscope (Evident, Waltham, MA, USA), and Nissl body density in the cerebral motor cortex was quantified using ImageJ software (version 1.41; National Institutes of Health, Bethesda, MD, USA).

### 2.13. Western Blot

Protein extracts obtained via RIPA lysis were analyzed via Western blot. Membranes were incubated overnight at 4 °C with primary antibodies targeting TLR4 (CST, 14358, 1:1000, Danvers, MA, USA), NFκB (Abcam, ab16502, 1:1000, Cambridge, UK), phospho-NFκB (CST, 3033, 1:1000, Danvers, MA, USA), Nrf2 (CST, 12721; 1:1000, Danvers, MA, USA), Caspase3 (Abcam, ab184787, 1:1000, Cambridge, UK), Cleaved-Caspase3 (Abcam, ab214430, 1:1000, Cambridge, UK), CD86 (Abmart, T55238, 1:1000, Shanghai, China), GAPDH (EpiZyme, LF205, 1:10000, Shanghai, China) and β-actin (Abcam, ab8226; 1:2000, Cambridge, UK). This was followed by 1-h incubation at RT with an HRP-conjugated goat anti-rabbit IgG secondary antibody (ZSGB-BIO, ZB-2301, 1:10,000, Beijing, China). Blots were developed with ECL reagent (Thermo, 34095, Waltham, MA, USA) and captured using a ChemiScope 6100 imaging system (Biolight, Guangzhou, China). For analysis of RAW264.7 cell lysates, GAPDH was used as the loading control. For the detection of Caspase3 and Cleaved-Caspase3 in lung epithelial cell lysates, β-actin was used as the loading control.

### 2.14. RT-qPCR

RNA was isolated from cells using the Super FastPure Cell RNA Isolation Kit (Vazyme, RC102-01, Nanjing, China) and subsequently reverse-transcribed into cDNA with HiScript III RT SuperMix for qPCR (Vazyme, R323-01, Nanjing, China). Quantitative real-time PCR was carried out in triplicate using Taq Pro Universal SYBR qPCR Master Mix (Vazyme, Q712-03, Nanjing, China) with 1–10 ng cDNA templates per reaction. Target gene expression levels were normalized to GAPDH and calculated using the 2^−ΔΔCt^ method. 2^−ΔΔCt^ method with normalization to GAPDH. The primer sequences employed for RT-qPCR are provided in Supplementary Table S1.

### 2.15. Immunofluorescent Staining

Following anesthesia via intraperitoneal injection of 0.25% sodium pentobarbital (three mice per group), brain specimens were embedded in optimal cutting temperature (OCT) medium (SAKURA, 4583, Tokyo, Japan) and subsequently sectioned at a thickness of 20 µm. The frozen sections, along with cultured cells, were fixed with 4% paraformaldehyde. The samples were further incubated at 4 °C overnight in a humidified chamber with primary antibodies to p-NFκB (rabbit, CST, 3033, Danvers, MA, USA), CD86 (rabbit, Abmart, T55238, Shanghai, China), and Iba-1 (rabbit, Abcam, ab178846, 1:1000, Cambridge, UK). Following careful washing, it was incubated with a secondary antibody (goat anti-rat IgG Alexa Fluor 488; Abcam, ab150077, Cambridge, UK) at room temperature (15–25 °C) for 60 min. Nuclei were stained with DAPI (Vectorlabs, H-1200-10, Newark, CA, USA) and imaged on a PerkinElmer UltraVIEW VOX confocal microscope (Waltham, MA, USA).

### 2.16. Three-Dimensional (3D) Structure Simulation and Molecular Docking

The peptide structure of rhTβ4 (GDT: 80.904) is based on AlphaFold2 modeling, and the tertiary structures of TLR4, NF-κB, and TNF-α are taken from the AlphaFold database. The separation of native ligands from protein structures, dehydration, and elimination of organic materials were performed via PyMOL (Version 3.1, Schrödinger, Inc., New York, NY, USA). Simulations of pairwise docking between the rhTβ4 peptide and the four proteins were subsequently performed through the HDOCK platform. Post-docking analysis, such as identifying intermolecular interactions and generating 3D/2D interaction plots, was carried out using Discovery Studio (4.0, Dassault Systèmes BIOVIA, San Diego, CA, USA) and LigPlus software (v2.2.8, EMBL-EBI, Hinxton, Cambridgeshire, UK).

### 2.17. RNA-Seq Assay

After the extraction of total RNA with Trizol reagent, mRNA was isolated by two rounds of Dynabeads Oligo (dT) selection. Paired-end sequencing (2 × 150 bp) was performed using sequencing libraries with a mean insert size of 300 + 50 bp on an Illumina NovaSeq™ 6000 system (LC-Bio Technology Co., Ltd., Hangzhou, China). Raw reads were filtered based on quality control with Cutadapt to produce a final clean dataset of G bp.

Data analysis began with read assembly and quantification for each sample using StringTie software (v2.2.1) with default parameters. Subsequently, gffcompare software (v0.12.6) was employed to integrate transcriptomes from all samples, constructing a comprehensive transcriptome. Based on the integrated transcriptome, transcript expression levels were quantified using StringTie and Ballgown (v2.36.0) and expressed as FPKM. Differentially expressed genes (DEGs) were identified using DESeq2 for inter-group comparisons and edgeR for paired-sample analyses. Genes with a false discovery rate (FDR) < 0.05 and an absolute fold change ≥ 2 were considered significantly differentially expressed.

### 2.18. Statistical Analysis

Statistical analyses were carried out using SPSS software (version 21.0; IBM Corp., Armonk, NY, USA). Continuous variables were presented as mean ± standard deviation (SD). Comparisons between groups were assessed via Student’s *t*-test or one-way ANOVA, with Tukey’s post hoc test applied for multiple comparisons. Survival data were analyzed using the Kaplan–Meier method. A two-tailed *p*-value < 0.05 was considered statistically significant for all tests.

## 3. Results

### 3.1. rhTβ4 Attenuates LPS-Induced Endotoxemia in a Mouse Model

**Figure 1:**
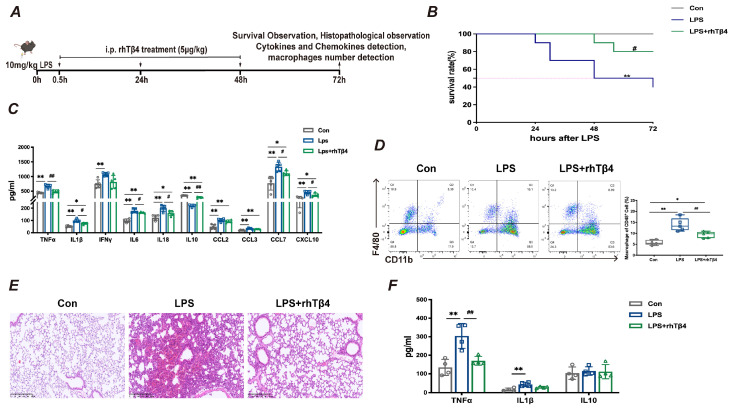
rhTβ4 attenuates LPS-induced ALI in mice. (**A**) Schematic of experimental timeline for the in vivo LPS-induced ALI model. Mice were injected intraperitoneally with LPS (10 mg/kg) and treated with rhTβ4 (5 μg/kg, i.p.) at 0.5 h after LPS challenge. (**B**) Kaplan–Meier survival curves of mice following LPS challenge with or without rhTβ4 treatment. LPS + rhTβ4 group exhibited an 80% survival rate at 72 h (*n* = 10). (**C**) Serum levels of TNF-α, IL-1β, IL-18, IL-6, IFN-γ, IL-10, CCL2, CCL3, CCL7, and CXCL10 measured by radioimmunoassay in the indicated groups (*n* = 5). (**D**) Flow cytometry analysis and quantitative summary of F4/80^+^ macrophage populations in lung tissues from Control, LPS-, and LPS + rhTβ4-treated mice (*n* = 5). (**E**) Representative H&E-stained lung sections from each group. Scale bar = 250 μm (*n* = 3). (**F**) Radioimmunoassay analysis of TNF-α, IL-1β, and IL-10 levels in lung homogenates from Control, LPS-, and LPS + rhTβ4-treated mice (*n* = 4). Abbreviations: TNF, tumor necrosis factor; IL, interleukin; IFN, interferon; CCL, C-C motif chemokine ligand; CXCL, C-X-C motif chemokine ligand. Differences with *p* < 0.05 were considered statistically significant. * *p* < 0.05, ** *p* < 0.01, vs. Con group. ^#^ *p* < 0.05, ^##^ *p* < 0.01, vs. LPS group.

To evaluate whether administration of rhTβ4 confers protective effects against endotoxemia-related mortality, we first assessed its therapeutic efficacy in a murine model of LPS-induced septic shock, as illustrated in the experimental schematic (Figure 1A). Treatment with rhTβ4 significantly improved survival outcomes, conferring an 80% survival rate at 72 h post-challenge and doubling the median survival time from 48 to 72 h (Figure 1B). Employing multiplex cytokine analysis, we demonstrated that rhTβ4 treatment led to a marked suppression of LPS-induced pro-inflammatory cytokine and chemokine elevations in plasma (Figure 1C) and lung tissue homogenates (Figure 1F). In addition, to determine whether rhTβ4 modulates macrophage recruitment to the lungs, we performed flow cytometric analysis on lung tissue samples. The results demonstrated that LPS challenge induced a substantial influx of macrophages into the pulmonary compartment, which was significantly attenuated by rhTβ4 treatment (Figure 1D). Consistent with these findings, histopathological evaluation via H&E staining confirmed that rhTβ4 treatment markedly mitigated LPS-induced inflammatory pathology, reducing inflammatory cell infiltration and alveolar damage (Figure 1E).

### 3.2. rhTβ4 Attenuated LPS-Induced Apoptosis of Alveolar Epithelial Cells

**Figure 2:**
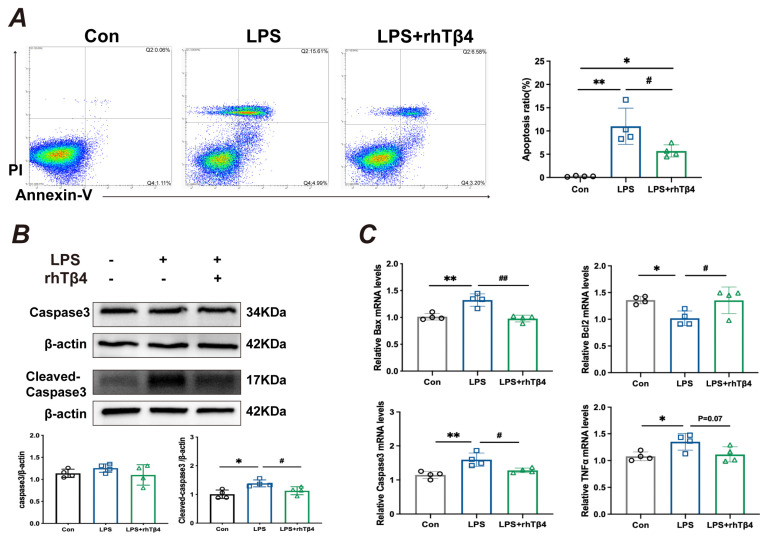
rhTβ4 attenuated LPS-induced apoptosis of alveolar epithelial cells. (**A**) Flow cytometry analysis and quantification of Con and LPS- and rhTβ4-treated RLE-6TN cells stained with Annexin V-FITC/PI (*n* = 4). (**B**) Western blot analysis of caspase3, cleaved caspase3 protein expression of Con, and LPS- and rhTβ4-treated RLE-6TN cells (*n* = 3). β-actin was used as the loading control. (**C**) RT-qPCR analysis of Bax, Bcl2, caspase3, and TNFα mRNA expression in RLE-6TN cells after LPS with or without rhTβ4 treatment (*n* = 4). Abbreviations: TNF, tumor necrosis factor; caspase3, Cysteine-aspartic acid protease 3; Bax, Bcl-2-associated X protein; Bcl2, B-cell lymphoma-2. Differences with *p* < 0.05 were considered statistically significant. * *p* < 0.05, ** *p* < 0.01, vs. Con group. ^#^ *p* < 0.05, ^##^ *p* < 0.01, vs. LPS group.

To test whether rhTβ4 inhibits LPS-induced apoptosis, we measured cell death in RLE-6TN alveolar epithelial cells pretreated with rhTβ4 and then stimulated with LPS. rhTβ4 markedly inhibited apoptosis of LPS-induced RLE-6TN cells, which was analyzed with Annexin V-FITC/PI by flow cytometry (Figure 2A). Furthermore, rhTβ4 significantly downregulated the mRNA expression of Bax and caspase3, which had been elevated by LPS, while it significantly upregulated the mRNA expression of Bcl2 in RLE-6TN cells (Figure 2C). Importantly, rhTβ4 effectively suppressed the key execution molecule of apoptosis, Cleaved caspase-3, likely inhibiting the apoptotic process (Figure 2B). These experimental results showed that rhTβ4 could inhibit apoptosis induced by LPS, which might serve as a mechanism of rhTβ4 alleviating ALI.

### 3.3. rhTβ4 Exerts a Concerted Suppression on the LPS-Induced Cytokine Cascade and Oxidative Stress

**Figure 3:**
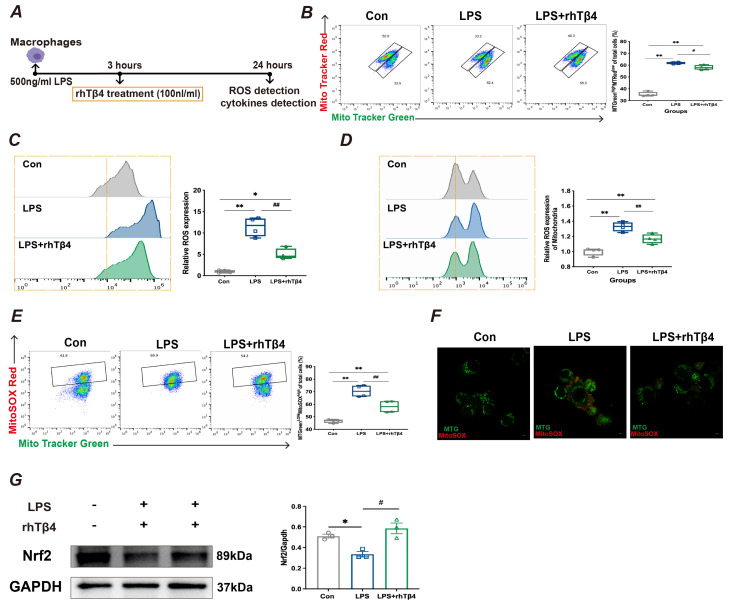
rhTβ4 alleviates oxidative stress in macrophages induced by LPS. (**A**) Experimental scheme of in vitro LPS stimulation and rhTβ4 treatment in RAW264.7 macrophages. RAW264.7 macrophages were stimulated with LPS (500 ng/mL), and rhTβ4 (100 ng/mL) was added 3 h after LPS stimulation. (**B**) Flow cytometry analysis and quantification of mitochondrial mass and membrane potential in RAW264.7 macrophages from Control, LPS-, and LPS + rhTβ4-treated groups, stained with MitoTracker Green (mass) and MitoTracker Red (membrane potential) (*n* = 4). (**C**,**D**) Flow cytometry analysis and quantification of cellular ROS levels using DCFH (**C**) and mitochondrial ROS levels using MitoSOX Red (**D**) in indicated groups (*n* = 4). (**E**) Flow cytometry analysis of RAW264.7 macrophages co-stained with MitoTracker Green and MitoSOX Red, with a quantitative summary of mitochondrial ROS-positive cells (*n* = 4). (**F**) Immunofluorescence images of RAW264.7 macrophages stained with MitoTracker Green (green) and MitoSOX Red (red). Scale bar = 5 μm. (**G**) Western blot analysis and quantitative summary of Nrf2 protein expression in macrophages from each group (*n* = 3). GAPDH was used as the loading control. Abbreviations: ROS, reactive oxygen species; MitoSox, Mitochondrial Superoxide Indicator; DCFH-DA, 2′,7′-dichlorodihydrofluorescein diacetate; Nrf2, nuclear factor erythroid 2-related factor 2; GAPDH, Glyceraldehyde-3-phosphate dehydrogenase. Differences with *p* < 0.05 were considered statistically significant. * *p* < 0.05, ** *p* < 0.01, vs. Con group. ^#^ *p* < 0.05, ^##^ *p* < 0.01, vs. LPS group.

The infiltration of macrophages leads to the production of elevated reactive oxygen species, which subsequently triggers an extensive pro-inflammatory cytokine cascade, thereby amplifying the local inflammatory response [24]. To evaluate the efficacy of rhTβ4 in attenuating LPS-induced oxidative stress and inflammation, we assessed mitochondrial homeostasis and ROS dynamics (Figure 3A). The combination of higher MitoTracker Green fluorescence (a measure of mitochondrial mass) and lower MitoTracker Red intensity (which indicates membrane potential) indicated that the stimulation of LPS caused depolarization of mitochondria. This depolarization was successfully abolished by rhTβ4 therapy (Figure 3B). In addition, rhTβ4 treatment reduced the overproduction of both cellular and mitochondrial ROS in response to LPS (Figure 3C,D). The results were verified via time-lapse imaging with MitoTracker Green and MitoSOX Red, which demonstrated that LPS induced an increase in mitochondrial subpopulations producing ROS, but this was prevented by rhTβ4 (Figure 3E,F). On the molecular level, the oxidative stress-associated transcription factor Nrf2 was studied, and it was found that LPS significantly inhibited its expression in macrophages, an effect that was strongly reversed by rhTβ4 (Figure 3G).

**Figure 4:**
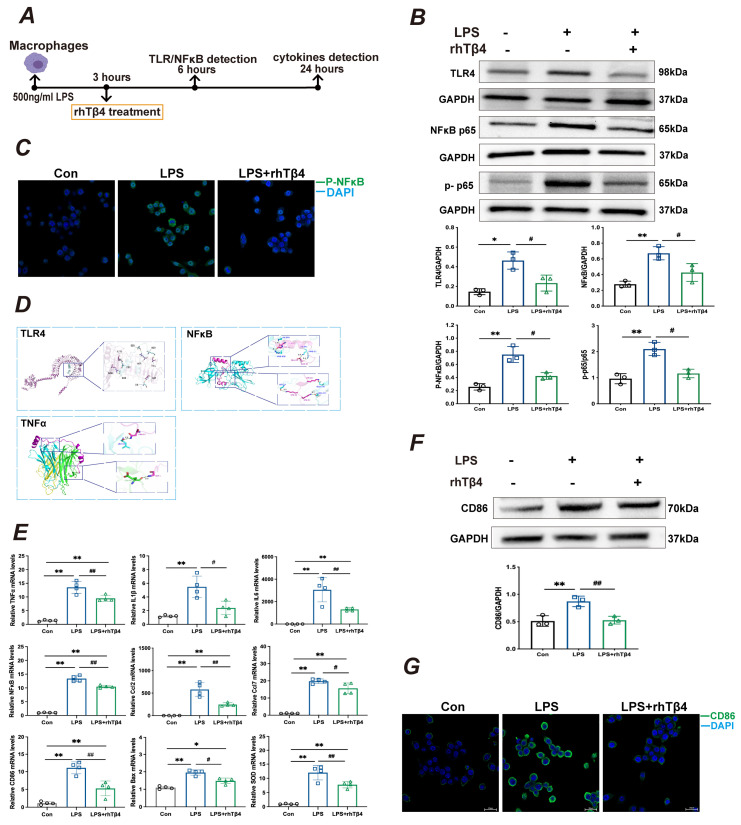
rhTβ4 suppresses the LPS-induced cytokine cascade via the TLR4/NF-κB pathway. (**A**) Experimental scheme of in vitro LPS stimulation and rhTβ4 treatment in RAW264.7 cells. RAW264.7 macrophages were stimulated with LPS (500 ng/mL), and rhTβ4 (100 ng/mL) was added 3 h after LPS stimulation. (**B**) Western blot analysis of TLR4, NF-κB p65, and phospho-NF-κB p65 protein expression in Control, LPS-, and LPS + rhTβ4-treated RAW264.7 macrophages. GAPDH was used as the loading control. (**C**) Representative immunofluorescence images showing the cellular localization of p-NF-κB p65 (green) in the indicated groups. Nuclei were counterstained with DAPI (blue). Scale bar = 20 µm. (**D**) Molecular docking models predicting the binding conformations of rhTβ4 with TLR4, NF-κB, and TNF-α. (**E**) RT-qPCR analysis of mRNA expression levels of TNF-α, IL-1β, IL-6, NF-κB, Ccl2, Ccl7, CD86, Bax, and SOD in RAW264.7 macrophages from the indicated groups (*n* = 4). (**F**) Western blot analysis of M1 macrophage marker CD86 protein expression in Control, LPS-, and LPS + rhTβ4-treated RAW264.7 macrophages (*n* = 3). GAPDH was used as the loading control. (**G**) Representative immunofluorescence images of CD86 (green) expression in RAW264.7 macrophages from the indicated groups. Scale bar = 20 µm. Abbreviations: TLR4, Toll-like receptor 4; NF-κB p65, nuclear factor-κB p65; p-NF-κB p65, phosphorylated NF-κB p65; DAPI, 4′,6-diamidino-2-phenylindole; CD86, Cluster of Differentiation 86; TNF, tumor necrosis factor; IL, interleukin; CCL, C-C motif chemokine ligand; SOD, Superoxide Dismutase; GAPDH, Glyceraldehyde-3-phosphate dehydrogenase; Bax, Bcl-2-associated X protein. Differences with *p* < 0.05 were considered statistically significant. * *p* < 0.05, ** *p* < 0.01, vs. Con group. ^#^ *p* < 0.05, ^##^ *p* < 0.01, vs. LPS group.

The dysregulated cytokine storm triggered by LPS, mediated through the TLR4/NF-κB axis, is a central driver of tissue damage and organ failure in endotoxemia [25]. We hypothesized that rhTβ4 exerts its therapeutic effects by intervening at this critical signaling node. The research we conducted using RAW264.7 cells challenged with LPS revealed that rhTβ4 has a strong inhibitory effect on the transcriptional upregulation of the major pro-inflammatory cytokines (TNF-α, IL-1 β, IL-6) and chemokines (Ccl2, Ccl7) (Figure 4D). This anti-inflammatory effect was rooted in the suppression of the upstream TLR4/NF-κB pathway, as evidenced by reduced TLR4 protein levels and inhibited NF-κB p65 phosphorylation (Figure 4B,C). An additional method used to examine possible interactions was molecular docking studies. The analysis showed possible modes of binding between rhTβ4 and key inflammatory mediators, such as TLR4, NF-κB, and TNF-α, with large calculated binding affinities (Figure 4D). These in silico predictions offer a structural justification that fits with our experimental findings and can also be used as a basis for further validation studies. In addition, rhTβ4 suppressed the polarization of M1 macrophages, which is the main phenotype of the pro-inflammatory reactions (Figure 4E,F). Hence, we infer that rhTβ4 exerts its protective effect through interaction with inflammation signaling molecules, thus terminating the cytokine cascade and macrophage polarization driven by NF-κB.

### 3.4. rhTβ4 Confers Protection Against Cognitive Dysfunction in Septic Mice by Mitigating Neuroinflammation and Neuronal Injury

**Figure 5:**
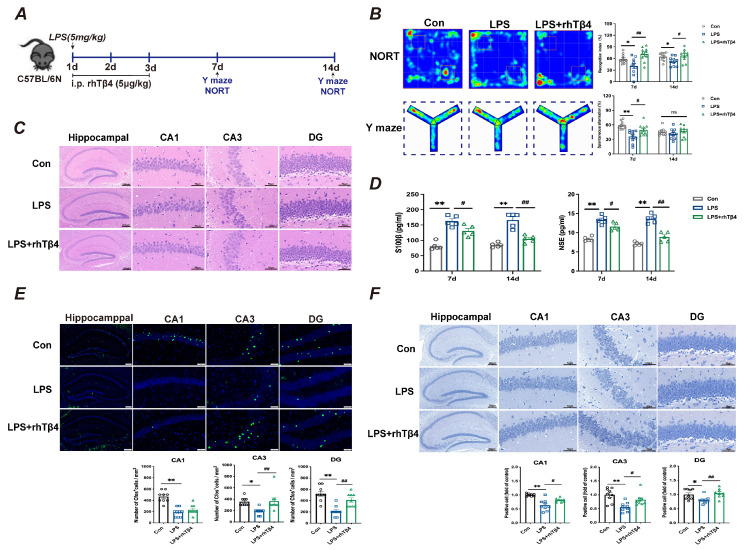
rhTβ4 alleviates LPS-induced memory impairment in mice with EAE. (**A**) Experimental timeline of the in vivo LPS-induced EAE model and behavioral testing. Mice were injected intraperitoneally with LPS (5 mg/kg) to induce EAE, and rhTβ4 (5 μg/kg) was administered at 0.5 h after LPS challenge. (**B**) Cognitive performance assessed by the novel object recognition test and Y-maze test in Control, LPS-, and LPS + rhTβ4-treated mice. (**C**) Representative H&E-stained sections of the hippocampal region from each group. Scale bars = 200 μm (overview) and 100 μm (magnified view) (*n* = 3). (**D**) Serum concentrations of neuronal injury markers S100β and NSE measured by radioimmunoassay (*n* = 5). (**E**) Representative c-fos immunofluorescence images (green) of the hippocampus of Control, and LPS- and rhTβ4-treated mouse brain. Scale bars = 200 μm (overview) and 50 μm (magnified view) (*n* = 3). (**F**) Representative Nissl-stained sections of the hippocampus of Control, and LPS- and rhTβ4-treated mouse brain. Scale bars = 200 μm (overview) and 100 μm (magnified view) (*n* = 3). Abbreviations: EAE, endotoxemia-associated encephalopathy; c-fos, cellular Fos proto-oncogene; NSE, neuron-specific enolase. Differences with *p* < 0.05 were considered statistically significant. * *p* < 0.05, ** *p* < 0.01, vs. Con group. ^#^ *p* < 0.05, ^##^ *p* < 0.01, vs. LPS group.

Cognitive decline is a major debilitating consequence of EAE, driven by LPS-induced neuroinflammation and subsequent neuronal damage [26]. We therefore investigated the therapeutic potential of rhTβ4 on memory function in an LPS-induced EAE model (Figure 5A). Behavioral tests confirmed that LPS administration induced significant cognitive impairment, marked by a decreased recognition index in the novel object recognition test and reduced spontaneous alternation in the Y-maze. Importantly, rhTβ4 treatment effectively reversed these behavioral abnormalities (Figure 5B). Corroborating these functional findings, histopathological examination showed a clear attenuation of LPS-induced brain damage in the rhTβ4-treated group (Figure 5C). The neuroprotective effect was further validated biochemically by a significant reduction in serum concentrations of the neuronal injury biomarkers S100β and NSE following rhTβ4 administration (Figure 5D). Nissl staining and c-fos immunofluorescence showed that rhTβ4 treatment robustly restored the damage to hippocampal neurons caused by LPS administration (Figure 5E,F). These findings establish that rhTβ4 treatment alleviates EAE-induced functional and pathological hallmarks, preserving memory and protecting against brain injury in mice.

### 3.5. rhTβ4 Alleviates Neuroinflammation Through the Suppression of Microglial Activation and Its Downstream Inflammatory Responses

**Figure 6:**
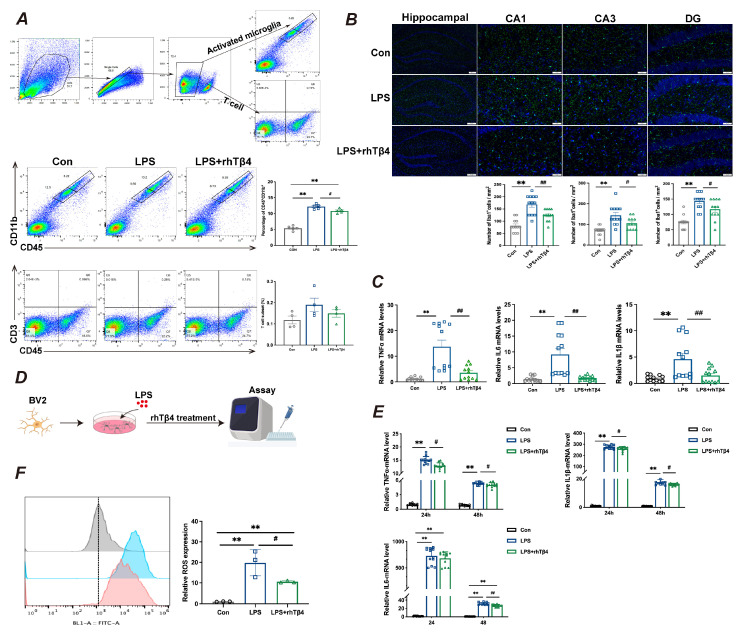
rhTβ4 suppresses microglial activation and neuroinflammation in mice. (**A**) Flow cytometry analysis and quantification of activated microglia (CD45^+^CD11b^high^) and T cells (CD3^+^) in brain homogenates from Control, LPS-, and LPS + rhTβ4-treated mice (*n* = 4). (**B**) Immunofluorescence images of brain sections stained with the microglial marker Iba-1 (green) from the indicated groups. Scale bars = 200 μm (overview) and 50 μm (magnified views) (*n* = 3). (**C**) RT-qPCR analysis of TNF-α, IL-1β, and IL-6 mRNA expression in brain tissues from Control, LPS-, and LPS + rhTβ4-treated mice (*n* = 4). (**D**) Experimental scheme of in vitro LPS stimulation and rhTβ4 treatment in BV2 microglial cells. BV-2 cells were stimulated with LPS (500 ng/mL) and treated with rhTβ4 (100 ng/mL) at 3 h after LPS stimulation. (**E**) RT-qPCR analysis of TNF-α, IL-1β, and IL-6 mRNA expression in BV2 microglia from the indicated groups (*n* = 4). (**F**) Flow cytometry analysis of intracellular ROS levels in BV2 microglia stained with DCFH-DA (*n* = 3). Abbreviations: CD45, Cluster of Differentiation 45; CD11b, Cluster of Differentiation 11b; CD3, Cluster of Differentiation 3; Iba-1, Ionized calcium-binding adapter molecule 1; TNF, tumor necrosis factor; IL, interleukin; ROS, reactive oxygen species; DCFH-DA, 2′,7′-dichlorodihydrofluorescein diacetate. Differences with *p* < 0.05 were considered statistically significant. ** *p* < 0.01, vs. Con group. ^#^ *p* < 0.05, ^##^ *p* < 0.01, vs. LPS group.

The activation of microglia is an important step in EAE, which causes the breakdown of the blood–brain barrier and promotes neuroinflammation, which in turn causes neuronal damage [27]. We therefore investigated whether rhTβ4 modulates this pathway. In LPS-treated mice, we observed a pronounced increase in activated microglia, which was reversed by rhTβ4 administration without affecting T cell numbers (Figure 6A,B). The suppression of microglial activation was accompanied by a marked downregulation of cerebral pro-inflammatory cytokines, including TNF-α, IL-6, and IL-1β (Figure 6C). Further validating its effects on microglia, rhTβ4 treatment in BV2 cells suppressed both the transcription of these inflammatory mediators and the associated oxidative stress, as evidenced by reduced ROS levels (Figure 6D–F). Together, these results indicate that rhTβ4 mitigates neuroinflammation, at least in part, by suppressing microglial activation and the associated inflammatory and oxidative responses.

### 3.6. rhTβ4 Suppresses Microglial Inflammation via LPAR3 Downregulation Identified by RNA-Seq Profiling

**Figure 7:**
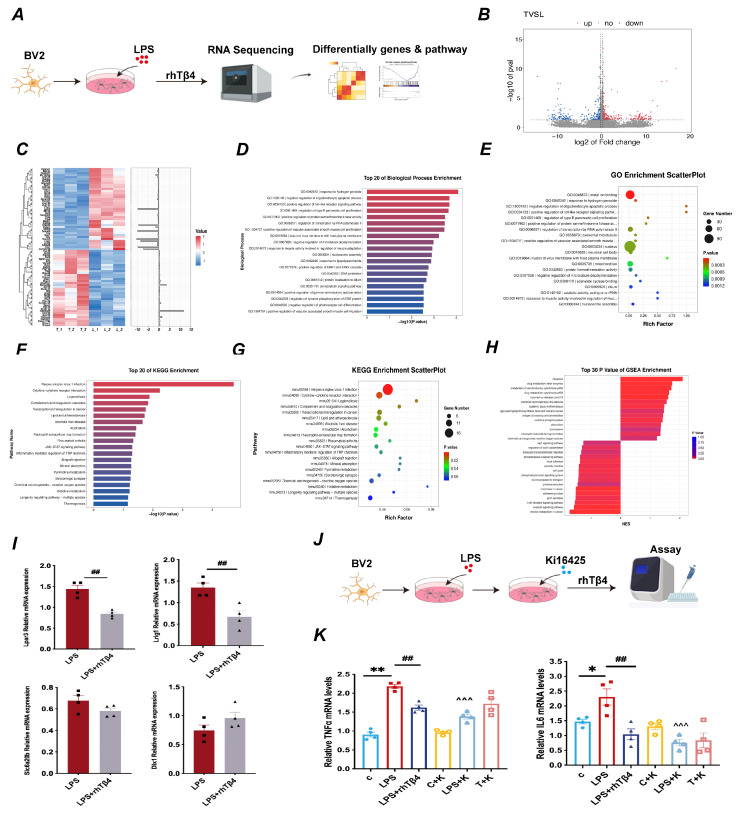
Transcriptomic profiling identifies LPAR3 downregulation as an important mechanism by which rhTβ4 attenuates microglial inflammation. (**A**) Schematic of the RNA-seq experimental design in BV2 microglia. BV-2 cells were stimulated with LPS (500 ng/mL) and treated with rhTβ4 (100 ng/mL) at 3 h after LPS stimulation. (**B**,**C**) Identification of DEGs in microglia following LPS and LPS+rhTβ4 treatment. (**B**) Volcano plot providing a graphical representation of significantly upregulated (red) and downregulated (blue) genes. (**C**) Heatmap depicting hierarchical clustering of DEGs. (**D**,**E**) Significantly enriched GO terms displayed as (**D**) bar plot and (**E**) bubble plot. (**F**,**G**) Significantly enriched KEGG pathways displayed as (**F**) bar plot and (**G**) bubble plot. (**H**) Top 30 GSEA profiles illustrating pathways negatively regulated by rhTβ4. (**I**) RT-qPCR validation of selected DEGs from RNA-Seq analysis (*n* = 3–4). (**J**) Experimental layout for pharmacological inhibition of LPAR3 in LPS-stimulated BV2 microglia. (**K**) RT-qPCR analysis of TNF-α and IL-6 mRNA levels in BV2 microglia treated with LPS, the LPAR3 antagonist Ki16425, and/or rhTβ4 (*n* = 4). Abbreviations: LPAR3, lysophosphatidic acid receptor 3; DEGs, differentially expressed genes; GO, Gene Ontology; KEGG, Kyoto Encyclopedia of Genes and Genomes; GSEA, Gene Set Enrichment Analysis; TNF, tumor necrosis factor; IL, interleukin; RNA-Seq, RNA sequencing. Differences with *p* < 0.05 were considered statistically significant. * *p* < 0.05, ** *p* < 0.01, vs. Con group. ^##^ *p* < 0.01, vs. LPS group. ^^^ *p* < 0.001, vs. C+K group.

To elucidate the mechanism by which rhTβ4 modulates microglial activity, we performed RNA-seq analysis on LPS-stimulated BV2 microglia with or without rhTβ4 treatment (Figure 7A). Comparative transcriptomics revealed 3558 DEGs, comprising 1899 downregulated and 1739 upregulated transcripts in rhTβ4-treated cells relative to the LPS group (Figure 7B). GO enrichment analysis indicated significant involvement of these DEGs in inflammatory response, apoptotic process, immune system process, and positive regulation of the Toll-like receptor pathway (Figure 7D,E). Concurrently, KEGG pathway analysis highlighted notable enrichment in necroptosis, cytokine-cytokine receptor interaction, PI3K-AKT signaling, and MAPK signaling pathways (Figure 7F,G). GSEA further demonstrated that rhTβ4 negatively regulated oxidative stress-related processes, the RAP1 signaling pathway, and leukocyte transendothelial migration, etc. (Figure 7H). A heatmap of DEGs illustrated that rhTβ4 counteracted the LPS-induced upregulation of several genes, including *Lpar3*, *Lrig1*, and *Dlx1*—a finding validated by RT-qPCR (Figure 7C,I). Among these, *Lpar3* exhibited the most pronounced differential expression. LPAR3 is a G protein-coupled receptor known to predominantly signal through Gq proteins [28]. Previous evidence suggests that LPAR3 activation can potentiate pro-inflammatory signaling cascades, including NF-κB [29]. To functionally validate the role of LPAR3, we applied Ki16425, a specific LPAR3 antagonist (Figure 7J). Ki16425 treatment significantly reduced TNF-α and IL-6 levels, effectively mimicking the anti-inflammatory effect of rhTβ4 (Figure 7K). These results suggest that downregulation of Lpar3 represents an important mechanism contributing to the anti-inflammatory action of rhTβ4.

## 4. Discussion

The consolidated findings of the present study demonstrate that rhTβ4 mediates potent therapeutic efficacy against LPS-induced endotoxemia and EAE via a multi-tiered mechanism entailing systemic immunomodulation and cell-level cytoprotective actions. In the LPS-induced endotoxemia model, rhTβ4 treatment elevated the 72-h survival rate to 80% and extended the median survival time. This protective effect was closely associated with its potent anti-inflammatory activity. rhTβ4 effectively suppressed macrophage recruitment into lung tissue and significantly reduced the levels of multiple pro-inflammatory cytokines and chemokines in both the circulation and local lung environment, thereby mitigating systemic inflammatory response syndrome and acute lung injury. The histopathological observations also supported its protection in regard to infiltration of inflammatory cells and damage to the tissues. These results suggest that by curbing the uncontrolled cytokine storm, rhTβ4 creates an important window for recovery following septic insult.

Tβ4 is a multifunctional peptide with the ability to induce tissue repair and orchestrate inflammatory reactions [30,31]. It has been shown to reduce neutrophil infiltration, change the levels of expression of CXCL1 and macrophage inflammatory protein-2, and control matrix metalloproteinase activity in order to maintain the integrity of the tissue [32]. In a recent Phase IIb study, it was shown that STEMI patients treated with intravenous rhTβ4 within 8 h after reperfusion had significantly smaller infarction areas and better ventricular remodeling. The study attributed these outcomes to activation of the ErbB2 signaling pathway [33]. This clinical evidence is supported by extensive preclinical studies demonstrating the efficacy of rhTβ4 in models of multi-organ injury, including neuroinflammation, pulmonary fibrosis, and impaired wound healing [30,32]. These protective properties are due to its pleiotropic anti-inflammatory, anti-apoptotic, and pro-regenerative properties. The recombinant derivative of native Tβ4, which is used in the current study, is rhTβ4 [34]. The protective efficacy of rhTβ4 stems from its concerted suppression of the canonical TLR4/NF-κB inflammatory axis and cellular oxidative stress. Activation of Toll-like and cytokine receptors causes a cascade of phosphorylation in which IκB kinase acts on IκB, leading to the release of NF-κB, its transport to the nucleus, and eventually to the activation of an inflammatory gene [35]. Our investigation revealed that rhTβ4 downregulated TLR4 protein expression and inhibited NF-κB p65 phosphorylation, thereby blocking the excessive activation of this pivotal signaling pathway upstream. Molecular docking simulations suggest possible binding conformations of rhTβ4 with TLR4, NF-κB, and TNF-α, although these computational predictions require biochemical validation (e.g., co-immunoprecipitation or surface plasmon resonance assays) to confirm physical interactions. Practically, this inhibition resulted in the inhibition of transcription of pro-inflammatory cytokines (e.g., TNF-α, IL-1β, IL 6) and reduction in M1 macrophage polarization. Simultaneously, rhTβ4 markedly alleviated LPS-induced dysfunction of mitochondria, restored the loss of mitochondrial membrane potential, and attenuated the production of both cellular and mitochondrial ROS. It seems that TLR4/NF-κB signaling and oxidative stress are part of a feedback amplification loop. On one hand, mitochondrial ROS (mtROS), which plays a vital role in amplifying the NF-κB activation process, participates in the early stages of its signaling, including the IκB degradation. On the other hand, NF-κB activation can contribute to oxidative stress by inducing the expression of the pro-oxidant enzymes and reducing the expression of the antioxidant regulator Nrf2. Our data suggest that rhTβ4 breaks this vicious circle by simultaneously suppressing both mtROS burst and NF-κB activation. Importantly, rhTβ4 also reversed the LPS-induced inhibition of Nrf2, further reducing oxidative stress and restoring cellular redox equilibrium. These results suggest that rhTβ4 does not achieve its protective effect through a single pathway, but rather through the simultaneous modulation of two interdependent core pathophysiological processes, inflammation (TLR4/NF-κB) and oxidative stress (Nrf2/ROS), and hence restores cellular homeostasis and alleviates the LPS-induced inflammatory injury.

Furthermore, an important advancement of this study is the discovery that rhTβ4 effectively prevents endotoxemia-associated cognitive dysfunction, with its neuroprotective effects appearing to be associated with the inhibition of microglial activation. One of the most crippling effects of EAE is cognitive impairment, yet effective pharmacological interventions remain scarce. Our behavioral measures, including NORT and the Y-maze spontaneous alternation test, collectively demonstrated that rhTβ4 treatment restored LPS-induced deficits in exploratory behavior, recognition memory, and spatial working memory significantly. These functional improvements indicate that rhTβ4 preserves cognitive integrity during systemic inflammatory challenge, a finding with substantial translational relevance given the persistent neurocognitive sequelae observed in sepsis survivors. In line with these behavioral findings, biochemical and histopathological analyses offered additional evidence of rhTβ4-mediated neuroprotection. Serum concentrations of NSE and S100β, established peripheral biomarkers of neuronal injury and blood–brain barrier disruption, were markedly elevated following LPS challenge and significantly reduced by rhTβ4 administration. These results suggest that rhTβ4 not only preserves neurons but also appears to maintain the integrity of the blood–brain barrier throughout systemic inflammation. These findings were supported by further histopathological analysis using Nissl staining, which showed attenuation of LPS-induced neuronal loss and morphological damage of the cerebral motor cortex and hippocampus brain areas that are essential in brain function. The maintenance of Nissl body density and neuronal architecture represents solid morphological support of the neuroprotective effectiveness of rhTβ4. Mechanistically, instead of regulating adaptive immune cell numbers, rhTβ4 exerts neuroprotective properties by inhibiting the activity of microglial cells. Flow cytometry of brain tissue revealed that the LPS challenge caused strong microglial activation, which was identified by enhanced Iba-1 positivity and changes in morphology that were heavily mitigated by rhTβ4 treatment. These changes in microglia were associated with functional outcomes: treatment with rhTβ4 significantly decreased levels of pro-inflammatory cytokines, such as TNF-α, IL-1β, and IL-6, in the brain-cytokines that are primarily secreted by microglia during the initial stage of neuroinflammation. Validation of the effect of rhTβ4 on the regulation of microglial function was achieved through in vitro experiments with the BV2 microglial cell culture. Stimulation of the BV2 cell culture with LPS reproduced the same inflammatory phenotype observed in vivo, which included high production of pro-inflammatory mediators and intracellular reactive oxygen species. Taken together, these findings indicate that rhTβ4 provides neuroprotection in endotoxemia, at least in part, by inhibiting microglial activation, which is accompanied by preserved cognitive function, reduced neuronal damage, and attenuated neuroinflammatory responses. However, we acknowledge that these data do not exclude potential effects of rhTβ4 on other cell types, and microglia, therefore, represent an important cellular target rather than the sole mediator of its neuroprotective effects.

Finally, a central mechanistic insight from this study, revealed by transcriptomic profiling, is that rhTβ4 suppresses microglial inflammation via downregulation of LPAR3. RNA-seq analysis and the subsequent validation indicated that rhTβ4 significantly reversed the LPS-induced upregulation of the Lpar3 gene. LPAR3 is a G protein-coupled receptor (GPCR) that predominantly associates with the Gαq/11 protein when activated by its ligand, LPA. Through activation of the canonical Gq-PLC-β pathway, this interaction leads to inositol trisphosphate (IP3) and diacylglycerol (DAG) production, culminating in intracellular calcium release and subsequent protein kinase C (PKC) activation [36]. PKC functions as a potent upstream kinase activating NF-κB, which in turn governs the transcription of pro-inflammatory mediators, notably TNF-α and IL-6 [29]. An experiment with a functional rescue with the specific LPAR3 antagonist Ki16425 replicated the anti-inflammatory action of rhTβ4, with a significant decrease in TNF-α and IL-6 levels. These results suggest that downregulation of LPAR3 is an important mechanism mediating the anti-inflammatory effects of rhTβ4 in microglia. However, we acknowledge that pharmacological inhibition alone does not definitively establish necessity or sufficiency, and we cannot exclude the potential contribution of alternative GPCR signaling pathways. LPAR3 thus represents an important, but not necessarily exclusive, mediator of these effects. Complementary genetic approaches will be required in future studies to further validate the central role of LPAR3 in this context.

It is well established that sex hormones, particularly estrogen, can significantly modulate inflammatory responses and influence the severity of endotoxemia [37,38]. Estrogen has been shown to suppress NF-κB activation, reduce the release of pro-inflammatory cytokines such as TNF-α and IL-6, potentially conferring a degree of endogenous protection in females during LPS-induced systemic inflammation. These sex-dependent immunological differences represent a potential limitation when extrapolating findings obtained exclusively from male animals to a broader population. To address this concern, we extended our investigation to include female mice and assessed the effects of rhTβ4 treatment on key inflammatory and survival outcomes (Supplementary Figure S1). Importantly, despite the potential immunomodulatory influence of estrogen on the baseline inflammatory milieu, rhTβ4 treatment resulted in significant attenuation of pro-inflammatory responses and alleviation of endotoxemia-associated organ injury in female mice, consistent with the effects observed in males. These findings support the candidacy of rhTβ4 as a sex-agnostic therapeutic intervention. Moreover, the clinical safety profile of rhTβ4 has been previously established in a wide range of therapeutic conditions such as cardiovascular disease, wound healing, and ophthalmic diseases [35]. These findings provide a foundation for repurposing rhTβ4 in endotoxemia—a condition characterized by the limited availability of therapies that can address the concurrent multi-organ dysfunction contributing to its morbidity and mortality. Unlike conventional approaches that often target single mediators or isolated pathways, the multifaceted pathophysiology of endotoxemia necessitates a therapeutic strategy capable of simultaneously modulating interconnected inflammatory cascades across multiple organ systems. Our findings suggest that rhTβ4 could serve as a candidate to address this unmet need. Moreover, the effectiveness of rhTβ4 in a variety of acute injury animal models of different severity, such as myocardial ischemia–reperfusion, corneal wound healing, and endotoxemia-induced lung and brain injury, indicates its potential as a multi-organ protector. While supportive care remains the mainstay of current clinical practice, interventions that actively limit the propagation of inflammatory injury across multiple organs are lacking. Based on our findings, we propose that rhTβ4 might serve as a therapeutic strategy to fill this gap by virtue of its coordinated inhibition of inflammatory signaling and oxidative stress. Collectively, these results, together with its established safety profile and sex-independent efficacy, suggest that rhTβ4 could be considered for further clinical investigation in endotoxemia-associated lung and brain dysfunction.

The limitations of this study should be acknowledged and addressed in future work. First, the pharmacological inhibition of LPAR3 with Ki16425 does not definitively establish its necessity, and the involvement of other GPCR pathways cannot be excluded. Complementary genetic approaches, such as conditional knockdown or knockout, will be required to unequivocally define the role of LPAR3 in future studies. Second, the molecular docking predictions require biochemical validation to confirm physical binding. We will use co-immunoprecipitation or pull-down assays to determine whether rhTβ4 physically interacts with TLR4, NF-κB, and TNF-αcellularly. Third, while our data demonstrate suppression of both TLR4 protein abundance and downstream NF-κB signaling, we acknowledge that the current experimental design does not allow definitive discrimination between transcriptional downregulation of TLR4 and inhibition of receptor activation. Both mechanisms may contribute to the observed anti-inflammatory phenotype, and elucidating their relative roles represents an important objective for future investigation. Collectively, these future investigations will be essential to validate the current mechanistic hypotheses and advance rhTβ4 toward clinical application.

## 5. Conclusions

This study indicates that rhTβ4 combats endotoxemia at the systemic level through the simultaneous inhibition of TLR4/NF-κB-mediated cytokine storms and cellular oxidative stress. Notably, our findings suggest that rhTβ4 mitigates neuroinflammation and subsequent cognitive impairment in EAE, at least in part, via downregulation of LPAR3 expression in microglia. These findings provide new insights into the biological functions of rhTβ4 and offer preclinical evidence that supports the development of therapeutic strategies against endotoxemia and EAE, combining anti-inflammatory and neuroprotective properties.

## Data Availability

The data that support the findings of this study are available from the corresponding author upon reasonable request. The data presented in the study are deposited in the GSA repository. https://ngdc.cncb.ac.cn/gsa/browse/CRA039814 (accessed on 13 March 2026). Accession number: CRA039814.

## Supplementary Materials

The following supporting information can be downloaded at https://www.mdpi.com/article/10.3390/biom16060766/s1, Table S1: RT-qPCR Primer Table. Figure S1. rhTβ4 attenuates LPS-induced Endotoxemia in female mice. (A) Kaplan–Meier survival curves of female mice following LPS challenge with or without rhTβ4 treatment. LPS + rhTβ4 group exhibited an 80% survival rate at 72 h (*n* = 10). (B) Flow cytometry analysis and quantification of F4/80^+^ macrophage populations in lung tissues from Control, LPS-, and LPS + rhTβ4-treated mice (*n* = 5). (C) RT-qPCR analysis of TNF-α and IL-6 mRNA expression in lung and brain tissues from Control, LPS-, and LPS + rhTβ4-treated mice (*n* = 4). (D) Flow cytometry analysis and quantification of activated microglia (CD45^+^CD11b^high^) in the brain from Control, LPS-, and LPS + rhTβ4-treated mice (*n* = 5). Abbreviations: TNF, tumor necrosis factor; IL, interleukin; CD45, Cluster of Differentiation 45; CD11b, Cluster of Differentiation 11b. Differences with *p* < 0.05 were considered statistically significant. * *p* < 0.05, ** *p* < 0.01, vs. Con group. ^#^ *p* < 0.05, ^##^ *p* < 0.01, vs. LPS group.
