# Supplementary material for: Recombinant Human Thymosin β4 Attenuates Endotoxemia-Induced ALI and EAE by Suppressing Inflammatory and Oxidative Responses
Source: Biomolecules. 2026 May 22;16(6):766. doi: 10.3390/biom16060766 (PMC13296540; doi:10.3390/biom16060766)
Supplement: Supplementary file 1 [file biomolecules-16-00766-s001.zip › biomolecules-4235899-supplementary.pdf]

Table S1. RT-qPCR Primer Table.

Primer	Sequence	Base number
TNFα-F	GCCTCTTCTCATTCCTGCTTGTG	23
TNFα-R	TGATGAGAGGGAGGCCATTTG	21
IL1β-F	CCAGGATGAGGACATGAGCAC	21
IL1β-R	TGTTGTTCATCTCGGAGCCTGTA	23
IL6-F	GTCGGAGGCTTAATTACACATGTTC	25
IL6-R	GCAAGTGCATCATCGTTGTTCA	22
NFκB-F	GGAGCAGGACATGGGATTTCA	21
NFκB-R	ACGGCCAGCAACATCTTCAC	20
CD86-F	ACGTATTGGAAGGAGATTACAGCT	24
CD86-R	TCTGTCAGCGTTACTATCCCGC	22
Bax-F	AGGATGCGTCCACCAAGAAGCT	22
Bax-R	TCCGTGTCCACGTCAGCAATCA	22
SOD-F	GGTGAACCAGTTGTGTTGTCAGG	23
SOD-R	ATGAGGTCCTGCACTGGTACAG	22
CCL7-F	TGCTCATAGCCGCTGCTTTC	20
CCL7-R	TTCTTGACATAGCAGCATGTGGA	23
CCL2-F	CAGCAGCAGGTGTCCCAAAG	20
CCL2-R	GATCTCATTTGGTTCCGATCCAG	23
Caspase3-F	ACGGTACGCGAAGAAAAGTGAC	22
Caspase3-R	GCTACACGCCTGCATCAGTA	20
Bcl2-F	TATATGGCCCCAGCATGCGA	20
Bcl2-R	GGGCAGGTTTGTCGACCTCA	20
Slc6a20b-F	GGCCACGTGGTGACTAAGGA	20

Primer	Sequence	Base number
Slc6a20bR	CTGATCTTGAAGTGGCGTGTGA	20
Dlx1-F	GAGGACCAATGAGCCTTGGAG	21
Dlx1-R	AGCCGCTGCTTGTGTCTTACTTTA	24
Lpar3-F	ATGCAGTAGGTAGGTGTACGCAAAG	25
Lpar3-R	GAGCAATAATGGCCCTCATCAAG	23
Lrig1-F	GGGACAGTGGCGGAACTATC	20
Lrig1-R	GATGGTATGGCTGTCAGCTCA	21
GAPDH-F	AGGTCGGTGTGAACGGATTTG	21
GAPDH-R	TGTAGACCATGTAGTTGAGGTCA	23

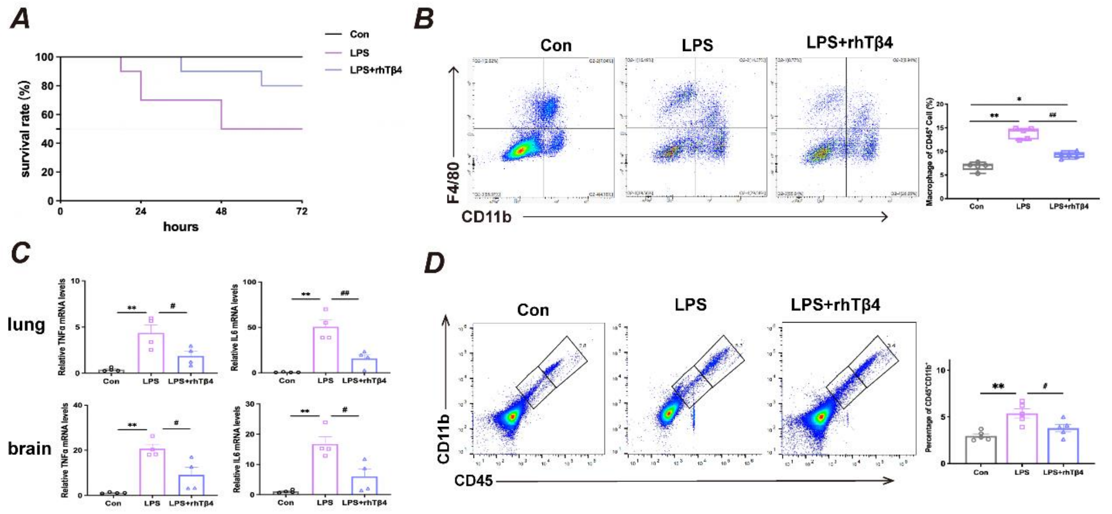

Figure S1. rhTβ4 attenuates LPS-induced Endotoxemia in female mice. (**A**) Kaplan–Meier survival curves of female mice following LPS challenge with or without rhTβ4 treatment. LPS + rhTβ4 group exhibited an 80% survival rate at 72 hours (n = 10). (**B**) Flow cytometry analysis and quantitative of F4/80+ macrophage populations in lung tissues from Control, LPS-, and LPS + rhTβ4-treated mice (n = 5). (**C**) RT-qPCR analysis of TNF-α and IL-6 mRNA expression in lung and brain tissues from Control, LPS-, and LPS + rhTβ4-treated mice (n = 4). (**D**) Flow cytometry analysis and quantification of activated microglia ($CD45^+$CD11bhigh) in brain from Control, LPS-, and LPS + rhTβ4-treated mice (n = 5). Abbreviations: TNF, tumor necrosis factor; IL, interleukin; CD45, Cluster of Differentiation 45; CD11b, Cluster of Differentiation 11b. Differences with P < 0.05 were considered statistically significant. *P < 0.05, ** $p < 0.01$, vs. Con group. # $p < 0.05$, ## $p < 0.01$, vs. LPS group.
